# Community health workers perspective on the COVID-19 impact on primary health care in Northeastern Brazil

**DOI:** 10.1590/0102-311XEN007223

**Published:** 2023-08-11

**Authors:** Anya Pimentel Gomes Fernandes Vieira-Meyer, Franklin Delano Soares Forte, José Maria Ximenes Guimarães, Sidney Feitoza Farias, André Luiz Sá de Oliveira, Maria Socorro de Araújo Dias, Claudete Ferreira de Souza Monteiro, Fernando José Guedes da Silva, Ana Patrícia Pereira Morais, Maria Rosilene Candido Moreira, Márcia C. Castro, Aisha Khizar Yousafzai

**Affiliations:** 1 Fundação Oswaldo Cruz, Eusébio, Brasil.; 2 Universidade Federal da Paraíba, João Pessoa, Brasil.; 3 Universidade Estadual do Ceará, Fortaleza, Brasil.; 4 Instituto Aggeu Magalhães, Fundação Oswaldo Cruz, Recife, Brasil.; 5 Universidade Estadual Vale do Acaraú, Sobral, Brasil.; 6 Universidade Federal do Piauí, Teresina, Brasil.; 7 Universidade Federal do Cariri, Crato, Brasil.; 8 Harvard TH Chan School of Public Health, Boston, U.S.A.

**Keywords:** Community Health Workers, Coronavirus Infection, COVID-19, Primary Health Care, Agentes Comunitarios de Saúde, Infecções por Coronavírus, COVID-19, Atenção Primária à Saúde, Agentes Comunitários de Salud, Infecciones por Coronavirus, COVID-19, Atención Primaria de Salud

## Abstract

This article evaluates the COVID-19 pandemic impacts on the Family Health Strategy (FHS) team’s work routines across a range of northeast Brazilian cities as perceived by community health workers (CHW). Data on COVID-19, CHW activities, and FHS teams were collected in 2021 by a structured questionnaire. A total of 1,935 CHWs from four state capitals (Fortaleza - Ceará State, João Pessoa - Paraíba State, Recife - Pernambuco State, Teresina - Piauí State) and four hinterland cities (Crato, Juazeiro do Norte, Barbalha, Sobral - Ceará State) participated in the study. Most CHWs were women (82.42%), with mean age 46.25±8.54 years. Many (39.92%) were infected with COVID-19, of which 70.78% believed they were infected in the workplace. A total of 77.82% defined their role as frontline in the fight against COVID-19, 16.07% reported receiving training for COVID-19, and 13.74% had access to sufficient protective equipment. Most (90.27%) believed their work routines were modified by the pandemic, either strengthening (41.46%) or weakening (44.41%) the team spirit. Home visits (60.55%), health promotion actions in schools (75.66%) and in specific community groups (93.96%), and other on-site community services (66.01%) showed a reduction in frequency. The sampled cities revealed a significant heterogeneity regarding responses to the COVID-19 pandemic, possibly associated with a lack of coordination by the Federal Government. Regardless of context, the pandemic led to a reconfiguration of local health systems, workflows, and primary care protocols for FHS teams. The importance of the Brazilian Unified National Health System (SUS) and its potential for reorganization during crisis should be acknowledged while preserving the headway made thus far.

## Resumo

Este artigo avalia as repercussões da pandemia da COVID-19 no cotidiano de trabalho da equipe da Estratégia Saúde da Família (ESF) em diversos municípios do Nordeste brasileiro, na perspectiva dos agentes comunitários de saúde (ACS). Um questionário estruturado foi utilizado para coletar informações sobre a COVID-19, atividades dos ACS e equipes da ESF em 2021. Participaram 1.935 ACS de quatro capitais (Fortaleza - Ceará, João Pessoa - Paraíba, Recife - Pernambuco e Teresina - Piauí) e quatro cidades do interior (Crato, Juazeiro do Norte, Barbalha e Sobral, Ceará). A idade média dos ACS era de 46,25±8,54 anos, sendo a maioria mulheres (82,42%). Muitos (39,92%) estavam infectados com COVID-19, dos quais 70,78% acreditavam ter sido infectados no ambiente de trabalho. Ao todo, 77,82% definiam seu papel como linha de frente no combate à COVID-19, 16,07% relataram receber treinamento para a COVID-19 e 13,74% tinham acesso a equipamentos de proteção suficientes contra a COVID-19. A maioria (90,27%) acredita que suas rotinas de trabalho foram modificadas pela pandemia, fortalecendo o espírito de equipe (41,46%) ou enfraquecendo-o (44,41%). Houve uma redução na promoção da saúde nas escolas (75,66%) e na frequência de visitas domiciliares (60,55%), de grupos específicos na comunidade (93,96%) e outros serviços comunitários locais (66,01%). Nos municípios avaliados, observou-se uma heterogeneidade significativa em relação à resposta à pandemia de COVID-19, possivelmente associada à falta de coordenação do Governo Federal. A pandemia levou a uma reconfiguração dos sistemas locais de saúde, fluxos de trabalho e protocolos de atenção primária para as equipes da ESF. A importância do Sistema Único de Saúde (SUS) e seu potencial de reorganização durante as crises devem ser reconhecidos, preservando-se os avanços alcançados até o momento.

## Resumen

Este artículo evalúa las repercusiones de la pandemia de COVID-19 en el trabajo cotidiano del equipo de la Estrategia Salud de la Familia (ESF) en diversos municipios del Nordeste brasileño, desde la perspectiva de los agentes comunitarios de salud (ACS). Se utilizó un cuestionario estructurado para recopilar información sobre COVID-19, actividades de los ACS y equipos de la ESF en el 2021. Participaron 1.935 ACS de cuatro capitales (Fortaleza - Ceará, João Pessoa - Paraíba, Recife - Pernambuco y Teresina - Piauí) y cuatro ciudades del interior (Crato, Juazeiro do Norte, Barbalha y Sobral - Ceará). La edad media de los ACS era de 46,25±8,54 años, y la mayoría eran mujeres (82,42%). Muchos (39,92%) estaban infectados con COVID-19, de los cuales el 70,78% creía haberse contagiado en el entorno laboral. En total, el 77,82% definió su papel como línea de frente en el combate a la COVID-19, el 16,07% informó haber recibido capacitación para la COVID-19 y el 13,74% tuvo acceso a equipos de protección suficiente contra la COVID-19. La mayoría (90,27%) cree que sus rutinas de trabajo se vieron modificadas por la pandemia, fortaleciendo el espíritu de equipo (41,46%) o debilitándolo (44,41%). Hubo una reducción en la promoción de la salud en las escuelas (75,66%) y en la frecuencia de las visitas domiciliarias (60,55%), de grupos específicos en la comunidad (93,96%) y otros servicios comunitarios locales (66,01%). En los municipios analizados, se observó una heterogeneidad significativa con relación a la respuesta a la pandemia de COVID-19, posiblemente asociada a la falta de coordinación del Gobierno Federal. La pandemia condujo a una reconfiguración de los sistemas locales de salud, los flujos de trabajo y los protocolos de atención primaria para los equipos de la ESF. Se debe reconocer la importancia del Sistema Único de Salud y (SUS) su capacidad de reorganización durante las crisis, preservando los avances logrados hasta el momento.

## Introduction

The COVID-19 pandemic constitutes one of the greatest healthcare challenges of the 21st century, heavily impacting healthcare systems, society, and science. Health care systems worldwide have proven to be inadequate to meet the crisis demands; but countries possessing a universal approach to public health care that cover health promotion, disease prevention and treatment of complex conditions, tend to be better prepared to combat pandemics ^1^. Brazilian Unified National Health System (SUS) is an important public health policy structured on the primary health care (PHC) model and implemented at the local level by the Family Health Strategy (FHS), which provides multiprofessional care. 

FHS is the country’s main strategy for consolidating and expanding primary care within the Brazilian National Primary Health Care Policy (PNAB/SUS), whose priorities include implementing democratic and participatory health care and management practices through multidisciplinary teams covering defined populations and territories, while considering each human being according to their singularity and socio-cultural context in a quest for comprehensive care ^2^. At the height of the pandemic, however, the SUS, which serves over 210 million Brazilians ^3^, was overburdened by the number of COVID-19 cases.

Epidemiological data available reflect the extension of the pandemic around the world. On April 19, 2023, the World Health Organization (WHO) registered 763,740,140 confirmed COVID-19 cases, associated with 6,908,554 deaths worldwide. In Brazil, the latest figures show 37,358,092 confirmed cases and 700,811 deaths, placing the country in third place globally in accumulated cases and second in deaths ^4^.

COVID-19 has aggravated social inequalities in Brazil. Not only did it produce more cases and deaths in poor regions, such as the North and Northeast, it also exacerbated poverty by increasing unemployment, sub-employment, and income loss. Northeastern Brazil accounts for 27% of the Brazilian population and registered one-third of COVID-19 cases (34%) and deaths (32%) in the early days of the pandemic, demonstrating that COVID-19 has taken a heavy toll on underdeveloped regions with profound social inequalities ^5^.

Experience over the past two years has pointed to the need for effective preparedness strategies to combat COVID-19, even a SUS reconfiguration. We should start by assessing the current system in which community health workers (CHW) play a crucial role ^6^. CHW integrate local FHS teams and possess detailed knowledge of the territories and populations they serve. Most of their work is performed in the territory where they live and includes providing guidance and support for their team’s health professionals and conducting home visits to collect information on the health and living standard of families. Their community presence facilitates residents’ access to health services and strengthens the bond between families and the FHS ^7^.

Their work is regulated by Brazilian legislation and play well-defined roles and specific activities as members of multiprofessional FHS teams. CHW assist families covered by the PHC system. Their work integrates a comprehensive and longitudinal health care framework that allows for the early identification of potentially severe cases ^8^ ^,^ ^9^.

During health emergencies, CHW can be deployed to educate the population on health promotion and disease prevention, and to help with contact tracking, post-treatment follow-up, and with identifying individuals requiring special health services. Their natural workplace allows them to enforce collective measures such as mask-wearing, quarantines, lockdowns, and agglomeration control ^10^.

Deploying the FHS, including CHW, improves health outcomes for a range of conditions and contexts ^11^ ^,^ ^12^ ^,^ ^13^ ^,^ ^14^. The sudden onset of the virus made additional CHW training and protection necessary, enabling them to efficiently detect and mitigate the COVID-19 spread while maintaining regular primary care services. Their contribution to public health during the pandemic has earned them a pivotal position in the workforce ^3^ ^,^ ^7^.

Given their territorial work and community-based approach, the experience of FHS workers in combating COVID-19 asserts the need for adapting work processes to logistic and spatial-temporal constraints, including new ways of delivering primary care services and addressing lockdown mandates during pandemics. Unfavorable changes in circumstances can limit transit between different areas of FHS coverage and weaken bonds with users and communities, especially by compromising home visit routines, health care flows, and the dynamic between team members (e.g., preventing monthly in-person planning meetings) ^8^ ^,^ ^15^.

Despite the eminently global nature of the COVID-19 pandemic, we must evaluate its impact on populations from cities of different sizes and socioeconomic profiles. Given the social, political, economic, and territorial inequalities of Northeastern Brazilian cities, virus exposure and its associated socioeconomic impacts affect social groups differently and to different degrees ^10^.

Community-based responses and territory-specific diagnoses are crucial to finding solutions that consider each region’s context and specific needs and avoid reproducing or exacerbating established inequalities ^10^. This study evaluated the repercussions of the COVID-19 pandemic on the work routines of FHS teams in different Northeastern Brazilian cities, as perceived by CHW.

## Methods

### Design and settings 

This cross-sectional multicenter study was conducted with data collected in four Northeastern state capitals (Fortaleza, Ceará State; João Pessoa, Paraíba State; Recife, Pernambuco State; Teresina, Piauí State) and in four municipalities (Crato, Juazeiro do Norte, Barbalha, Sobral - Ceará State) in the hinterland of Ceará. Selection criteria for the state capitals included variation on city size, FHS and Community Health Agents Program coverage, as well as morbidity and mortality due to COVID-19 indicators. As for the municipalities, we added the criteria of being a metropolitan region removed from the capital. Following the STROBE (Strengthening the Reporting of Observational Studies in Epidemiology) guidelines ^16^, the research was carried out by researchers from 13 different institutions located in the chosen municipalities.

### Population and sampling

A simple, random CHW sample was built for each city, with a 5% sampling error, 95% confidence interval (95%CI), and homogenous distribution (80/20). Eligibility criteria included CHWs currently active on the job. CHW on vacation or sick leave were not eligible (Table 1).

**Table 1:** Number of active community health workers (CHW) per city and sample size calculated per municipality.

City	Active CHWs	Calculated sample *
Fortaleza	2,119	364
Recife	1,899	320
João Pessoa	1,368	303
Teresina	1,320	309
Sobral	415	203
Barbalha	121	93
Juazeiro do Norte	483	215
Crato	184	127
Total	7,909	1,935

Source: Brazilian Ministry of Health ^31^.

* A random sample calculation was built for each city considering a 5% sampling error, 95% confidence interval, and homogenous distribution (80/20).

### Data collection

Prior to data collection, we obtained authorization from the respective municipal officials, and scheduled visits to the selected FHS facilities for questionnaire application. All participants completed the questionnaires in the presence of a data collector, which allowed to clarify doubts as they arose. Data collectors underwent a 12-hour training program (e.g., research project, quantitative data collection, biosafety protocols, ethical aspects of research involving humans, and data collection instruments) before initiating the fieldwork. The research instruments collected sociodemographic data (e.g., gender, marital status, race/ethnicity, schooling, income), information on experience with COVID-19 (e.g., coworker has/had COVID-19, relatives have/had COVID-19, respondent had COVID-19, severity of respondent COVID-19 infection, respondent believes to have been infected at work), the pandemic impact on the CHW’s abilities (e.g., identifies as a COVID-19 frontline worker, received COVID-19 related training, considers conventional personal protective equipment (PPE) efficient for COVID-19, workplace availability of sufficient PPE for COVID-19 related activities, considers workplace safety COVID-19 norms to be sufficient, consider the workplace a risk of COVID-19 infection, work routines changed by the pandemic, teamwork was affected by the pandemic, changes in activity planning, frequency of home visits during the pandemic, frequency of Brazilian School Health Program (PSE) during the pandemic, frequency of specific group activities during the pandemic, frequency of other on-site community services during the pandemic, frequency of office work during the pandemic), and the FHS team to provide primary care (e.g., COVID-19 impact on primary care provision, frequency of care for diabetes mellitus and arterial hypertension, frequency of antenatal care, frequency of vaccination, frequency of care for tuberculosis, frequency of care for leprosy, frequency of infant care, frequency of care for spontaneous demands, frequency of family planning, frequency of cervical cancer screening, frequency of rapid testing, frequency of pregnancy testing, and frequency of oral health appointments). Data were collected between April and August 2021. All biosafety guidelines outlined in the *GVIM/GGTES/ANVISA n. 04/2020 Technical Note* were followed ^17^.

### Data analysis

All data analyses were performed using Stata 14.0 (https://www.stata.com). Quantitative data were entered into a digital spreadsheet. Nominal variables were expressed as absolute and relative frequencies, whereas quantitative variables were expressed as mean values ± standard deviation. Independence of the explanatory variables was verified by chi-square test. Magnitudes of statistically significant associations (p < 0.05) were expressed as prevalence ratios. Statistical significance level was set at 5%.

### Ethical consideration

All participants voluntarily agreed to participate in the study after explanations about the study and completing the online consent form. The study protocol was approved by the Ceará State University’s (UECE) ethics committee and filed under n. 4,587,955. 

## Results

A total of 1,935 CHWs answered the questionnaire, distributed between eight cities: Fortaleza (n = 364), João Pessoa (n = 303), Recife (n = 320), Teresina (n = 309), Sobral (n = 203), Juazeiro do Norte (n = 215), Crato (n = 127) and Barbalha (n = 93). Mean age was 46.25±8.54 years, with the lowest average in Sobral (43.01±10.27) and the highest in Juazeiro do Norte (47.20±8.14). Most CHW were female (n = 1,594; 82.42%), married/partnered (n = 1,130; 58.4%), Catholic (n = 1,122; 63.15%), mixed race (n = 1,389; 71.93%), with compete high school education (n = 1,112; 57.47%), and in the 1-2 minimum wage income bracket (n = 985; 54.87%). Overall sociodemographic profile varied significantly between cities (Table 2).

**Table 2:** Community health workers (CHW) sociodemographic data and experience with COVID-19 per city (N = 1,935).

	Fortaleza n (%)	João Pessoa n (%)	Recife n (%)	Teresina n (%)	Sobral n (%)	Juazeiro do Norte n (%)	Crato n (%)	Barbalha n (%)	p-value	Total n (%)
Sociodemographic characteristics										
Gender [n = 1,934]										
Male	63 (17.31)	65 (21.45)	39 (12.19)	95 (30.74)	23 (11.33)	25 (11.63)	11 (8.66)	19 (20.43)	< 0.001	340 (17.58)
Female	301 (82.69)	238 (78.55)	281 (87.81)	214 (69.26)	180 (88.67)	190 (88.37)	116 (91.34)	74 (79.57)		1,594 (82.42)
Marital status [n = 1,935]										
Married/Partnered	205 (56.32)	158 (52.15)	163 (50.78)	207 (66.99)	121 (59.61)	127 (59.07)	83 (65.35)	66 (70.97)	0.001	1,130 (58.40)
Separated/Divorced	52 (14.29)	45 (14.85)	42 (13.08)	38 (12.30)	18 (8.87)	31 (14.42)	13 (10.24)	10 (10.75)		249 (12.87)
Single	101 (27.75)	90 (29.70)	100 (31.15)	54 (17.48)	58 (28.57)	47 (21.86)	29 (22.83)	14 (15.05)		493 (25.48)
Widowed	6 (1.65)	10 (3.30)	16 (4.98)	10 (3.24)	6 (2.96)	10 (4.65)	2 (1.57)	3 (3.23)		63 (3.26)
Race/Ethnicity [n = 1,931]										
Caucasian/Asian	44 (12.15)	61 (20.20)	34 (10.59)	15 (4.85)	16 (7.88)	48 (22.33)	22 (17.46)	10 (10.75)	< 0.001	250 (12.95)
Brown/Indigenous/Mixed	278 (76.80)	196 (64.90)	214 (66.67)	234 (75.73)	161 (79.31)	140 (65.12)	90 (71.43)	76 (81.72)		1,389 (71.93)
Black	40 (11.05)	45 (14.90)	73 (22.74)	60 (19.42)	26 (12.81)	27 (12.56)	14 (11.11)	7 (7.53)		292 (15.12)
Schooling [n = 1,935]										
Complete elementary school	30 (8.24)	27 (8.91)	43 (13.40)	12 (3.88)	12 (5.91)	20 (9.30)	14 (11.02)	3 (3.23)	< 0.001	161 (8.32)
Complete high school	216 (59.34)	164 (54.13)	190 (59.19)	186 (60.19)	148 (72.91)	99 (46.05)	71 (55.91)	38 (40.86)		1,112 (57.47)
Complete tertiary education	118 (32.42)	112 (36.96)	88 (27.41)	111 (35.92)	43 (21.18)	96 (44.65)	42 (33.07)	52 (55.91)		662 (34.21)
Income (MW) [n = 1,795]										
< 1	20 (6.13)	14 (5.04)	24 (7.77)	14 (4.83)	9 (4.48)	6 (3.17)	6 (5.22)	5 (5.75)	< 0.001	98 (5.46)
≥ 1 to < 2	179 (54.91)	131 (47.12)	166 (53.72)	160 (55.17)	138 (68.66)	105 (55.56)	65 (56.52)	41 (47.13)		985 (54.87)
2-3	101 (30.98)	99 (35.61)	100 (32.36)	85 (29.31)	48 (23.88)	69 (36.51)	36 (31.30)	27 (31.03)		565 (31.48)
≥ 4	26 (7.98)	34 (12.23)	19 (6.15)	31 (10.69)	6 (2.99)	9 (4.76)	8 (6.96)	14 (16.09)		147 (8.19)
Experience with COVID-19 infection										
Coworker has/had COVID-19 [n = 1,869]										
No	10 (2.85)	3 (1.02)	9 (2.93)	15 (5.08)	7 (3.57)	14 (6.70)	15 (11.90)	7 (7.61)	0.003	80 (4.35)
Yes	334 (95.16)	285 (97.27)	295 (96.09)	277 (93.90)	187 (95.41)	191 (91.39)	107 (84.92)	82 (89.13)		1,758 (95.65)
Not sure	7 (1.99)	5 (1.71)	3 (0.98)	3 (1.02)	2 (1.02)	4 (1.91)	4 (3.17)	3 (3.26)		31 (1.66)
Relatives have/had COVID-19 [n = 1,871]										
No	63 (17.80)	62 (21.16)	71 (23.13)	87 (29.49)	46 (23.47)	52 (25.00)	34 (26.98)	28 (30.43	0.020	443 (23.68)
Yes	291 (82.20)	231 (78.84)	236 (76.87)	208 (70.51)	150 (76.53)	156 (75.00)	92 (73.02)	64 (69.57)		1,428 (76.32)
Respondent had COVID-19 [n = 1,934]										
No	193 (53.02)	184 (60.93)	176 (54.83)	224 (72.49)	115 (56.65)	124 (57.67)	80 (62.99)	66 (70.97)	< 0.001	1,162 (60.08)
Yes	171 (46.98)	118 (39.07)	145 (45.17)	85 (27.51)	88 (43.35)	91 (42.33)	47 (37.01)	27 (29.03)		772 (39.92)
Severity of respondent COVID-19 infection [n = 770]										
Mild	56 (32.75)	37 (31.90)	44 (30.34)	43 (50.59)	41 (46.59)	38 (41.76)	25 (53.19)	11 (40.74)	0.002	295 (38.31)
Moderate	85 (49.71)	63 (54.31)	80 (55.17)	37 (43.53)	41 (46.59)	48 (52.75)	15 (31.91)	9 (33.33)		378 (49.09)
Severe	27 (15.79)	16 (13.79)	19 (13.10)	5 (5.88)	5 (5.68)	4 (4.40)	6 (12.77)	5 (18.52)		87 (11.30)
style="text-indent:12px"Very severe	3 (1.75)	-	2 (1.38)	-	1 (1.14)	1 (1.10)	1 (2.13)	2 (7.41)		10 (1.30)
Respondent believes to have been infected at work [n = 770]										
No	70 (40.94)	43 (37.07)	59 (40.69)	50 (58.82)	45 (51.14)	48 (52.75)	26 (55.32)	12 (44.44)	< 0.001	225 (29.22)
Yes	101 (59.06)	73 (62.93)	86 (59.31)	35 (41.18)	43 (48.86)	43 (47.25)	21 (44.68)	15 (55.56)		545 (70.78)
Respondent has sequelae from COVID-19 [n = 770]										
No	70 (40.94)	43 (37.07)	59 (40.69)	50 (58.82)	45 (51.14)	48 (52.75)	26 (55.32)	12 (44.44)	0.017	353 (45.84)
Yes	101 (59.06)	73 (62.93)	86 (59.31)	35 (41.18)	43 (48.86)	43 (47.25)	21 (44.68)	15 (55.56)		417 (54.16)

MW: minimum wage (corresponding to USD 230 at the time of writing).

### COVID-19 infection among CHWs

Most participants reported having been infected with COVID-19 (n = 772, 39.92%). Of these, 475 (61.69%) considered the infection either moderate, severe, or very severe, whereas 417 (54.16%) reported having sequelae. Over two-thirds of the infected CHW (n = 545, 70.78%) believed they contracted the virus at work. Table 2 summarizes the respondents’ experience with COVID-19 infection. Most (n = 1,758, 95.65%) reported having a coworker who was infected. Inter-city difference in the experience with COVID-19 infection are noteworthy, varying from 97.27% in João Pessoa to 84.92% in Crato. 

### CHW as COVID-19 frontline workers

A total of 1,502 respondents (77.82%) self-identified as COVID-19 frontline workers, but only 309 (16.07%) reported receiving training to work in this setting. A total of 1,320 (79.18%) believe the workplace safety procedures adopted were insufficient for their protection, and very few (n = 265; 13.74%, with cities ranging between 4.19% and 31.03%) confirmed a sufficient supply of PPE (including masks and face shields). Table 3 shows the differences in CHW preparedness between the sampled cities. Major discrepancies can be observed between Sobral and João Pessoa (COVID-19 training 42.36% vs 6.69%; availability of sufficient PPE 31.03% vs 3.63%).

**Table 3:** Community health workers (CHW) preparedness for COVID-19 and perceived impact of COVID-19 on their work routine/activities per city (N = 1,935).

	Fortaleza n (%)	João Pessoa n (%)	Recife n (%)	Teresina n (%)	Sobral n (%)	Juazeiro do Norte n (%)	Crato n (%)	Barbalha n (%)	p-value	Total n (%)
COVID										
Identifies as a COVID-19 frontline worker [n = 1,930]										
No	55 (15.24)	26 (8.64)	115 (35.83)	146 (47.25)	3 (1.48)	58 (26.98)	21 (16.54)	4 (4.30)	< 0.001	428 (22.18)
Yes	306 (84.76)	275 (91.36)	206 (64.17)	163 (52.75)	200 (98.52)	157 (73.02)	106 (83.46)	89 (95.70)		1,502 (77.82)
Received COVID-19-related training [n = 1,923]										
No	302 (84.36)	279 (93.31)	288 (90.28)	258 (83.50)	117 (57.64)	181 (84.19)	114 (89.76)	75 (80.65)	< 0.001	1,614 (83.93)
Yes	56 (15.64)	20 (6.69)	31 (9.72)	51 (16.50)	86 (42.36)	34 (15.81)	13 (10.24)	18 (19.35)		309 (16.07)
Considers conventional PPE efficient for COVID-19 [n = 1,930]										
No	155 (42.82)	185 (61.46)	165 (51.56)	128 (41.42)	101 (49.75)	109 (50.70)	58 (45.67)	50 (53.76)	< 0.001	951 (49.27)
Yes	207 (57.18)	116 (38.54)	155 (48.44)	181 (58.58)	102 (50.25)	106 (49.30)	69 (54.33)	43 (46.24)		979 (50.73)
Availability of sufficient PPE in the workplace for COVID-19-related activities [n = 1,929]										
No	306 (85.24)	292 (96.37)	279 (87.19)	255 (82.52)	140 (68.97)	206 (95.81)	104 (81.89)	82 (88.17)	< 0.001	1,664 (86.26)
Yes	53 (14.76)	11 (3.63)	41 (12.81)	54 (17.48)	63 (31.03)	9 (4.19)	23 (18.11)	11 (11.83)		265 (13.74)
Considers safety norms at work to be sufficient for COVID-19 [n = 1,934]										
No	241 (66.21)	262 (86.75)	234 (72.90)	185 (59.87)	99 (48.77)	153 (71.16)	75 (59.06)	71 (76.34)	< 0.001	1,320 (79.18)
Yes	67 (18.41)	20 (6.62)	53 (16.51)	62 (20.06)	86 (42.36)	21 (9.77)	23 (18.11)	15 (16.13)		267 (13.81)
Don’t know	56 (15.38)	20 (6.62)	34 (10.59)	62 (20.06)	18 (8.87)	41 (19.07)	29 (22.83)	7 (7.53)		347 (20.82)
Consider the workplace a risk of COVID-19 infection [n = 1,933]										
No	9 (2.47)	12 (3.99)	10 (3.12)	11 (3.56)	7 (3.45)	5 (2.33)	4 (3.15)	3 (3.23)	< 0.001	61 (3.33)
Yes	343 (94.23)	282 (93.69)	292 (90.97)	266 (86.08)	193 (95.07)	199 (92.56)	108 (85.04)	88 (94.62)		1,771 (96,67)
Not sure	12 (3.30)	7 (2.33)	19 (5.92)	32 (10.36)	3 (1.48)	11 (5.12)	15 (11.81)	2 (2.15)		101 (5.23)
Impact of COVID-19 on CHW work routine										
Work routines changed by the pandemic [n = 1,932]										
No	51 (14.09)	26 (8.61)	35 (10.90)	24 (7.77)	9 (4.43)	21 (9.77)	11 (8.66)	11 (11.83)	0.016	188 (9.73)
Yes	311 (85.91)	276 (91.39)	286 (89.10)	285 (92.23)	194 (95.57)	194 (90.23)	116 (91.34)	82 (88.17)		1,744 (90.27)
Teamwork was affected by the pandemic [n = 1,763]										
No	57 (17.07)	33 (11.66)	45 (14.85)	38 (13.43)	14 (7.25)	30 (17.24)	17 (15.74)	15 (17.65)	< 0.001	249 (14.12)
Yes, the team spirit was weakened	108 (32.34)	115 (40.64)	147 (48.51)	71 (25.09)	140 (72.54)	92 (52.87)	68 (62.96)	42 (49.41)		783 (44.41)
Yes, the team spirit was strengthened	169 (50.60)	135 (47.70)	111 (36.63)	174 (61.48)	39 (20.21)	52 (29.89)	23 (21.30)	28 (32.94)		731 (41.46)
Changes in activity planning [n = 1,760]										
No	34 (10.21)	15 (5.24)	26 (8.72)	24 (8.42)	6 (3.08)	16 (9.36)	9 (8.33)	10 (11.90)	< 0.001	140 (7.95)
Yes, planning improved	88 (26.43)	80 (27.97)	121 (40.60)	59 (20.70)	121 (62.05)	77 (45.03)	40 (37.04)	36 (42.86)		622 (35.34)
Yes, planning worsened	211 (63.36)	191 (66.78)	151 (50.67)	202 (70.88)	68 (34.87)	78 (45.61)	59 (54.63)	38 (45.24)		998 (56.70)
Frequency of home visits during the pandemic [n = 1,929]										
No change	174 (48.47)	52 (17.16)	64 (19.94)	51 (16.56)	40 (19.70)	62 (28.84)	40 (31.50)	28 (30.11)	< 0.001	511 (26.49)
Lower	125 (34.82)	242 (79.87)	243 (75.70)	244 (79.22)	113 (55.67)	119 (55.35)	45 (35.43)	37 (39.78)		1,168 (60.55)
Higher	60 (16.71)	9 (2.97)	14 (4.36)	13 (4.22)	50 (24.63)	34 (15.81)	42 (33.07)	28 (30.11)		250 (12.96)
Frequency of Brazilian School Health Program during the pandemic [n = 1,903]									< 0.001	
No change	14 (4.01)	11 (3.78)	7 (2.19)	14 (4.58)	7 (3.45)	21 (9.77)	12 (9.45)	6 (6.45)		330 (17.13)
Lower	326 (93.41)	279 (95.88)	312 (97.81)	289 (94.44)	194 (95.57)	191 (88.84)	113 (88.98)	84 (90.32)		1,458 (75.66)
Higher	9 (2.58)	1 (0.34)	0 (0.00)	3 (0.98)	2 (0.99)	3 (1.40)	2 (1.57)	3 (3.23)		139 (7.21)
Frequency of group-specific activities during the pandemic [n = 1,927]										
No change	63 (17.50)	41 (13.62)	47 (14.69)	66 (21.43)	15 (7.39)	50 (23.26)	24 (18.90)	24 (25.81)	< 0.001	92 (4.83)
Lower	268 (74.44)	248 (82.39)	251 (78.44)	227 (73.70)	169 (83.25)	150 (69.77)	92 (72.44)	53 (56.99)		1,788 (93.96)
Higher	29 (8.06)	12 (3.99)	22 (6.88)	15 (4.87)	19 (9.36)	15 (6.98)	11 (8.66)	16 (17.20)		23 (1.21)
Frequency of other on-site community services during the pandemic [n = 1,927]										
No change	33 (9.14)	73 (24.33)	69 (21.50)	37 (12.05)	40 (19.70)	52 (24.19)	26 (20.47)	27 (29.03)	< 0.001	357 (18.53)
Lower	289 (80.06)	180 (60.00)	212 (66.04)	246 (80.13)	125 (61.58)	128 (59.53)	65 (51.18)	27 (29.03)		1,272 (66.01)
Higher	39 (10.80)	47 (15.67)	40 (12.46)	24 (7.82)	38 (18.72)	35 (16.28)	36 (28.35)	39 (41.94)		298 (15.46)
Frequency of office work during the pandemic [n = 1,909]										
No change	128 (37.32)	185 (61.67)	136 (42.37)	113 (36.81)	105 (51.72)	107 (49.77)	69 (54.33)	53 (56.99)	< 0.001	896 (46.94)
Lower	142 (41.40)	51 (17.00)	122 (38.01)	168 (54.72)	28 (13.79)	70 (32.56)	18 (14.17)	6 (6.45)		605 (31.69)
Higher	73 (21.28)	64 (21.33)	63 (19.63)	26 (8.47)	70 (34.48)	38 (17.67)	40 (31.50)	34 (36.56)		408 (21.37)

PPE: personal protective equipment.

### Perceived impact of COVID-19 on PHC work performance

Tables 3 and 4 summarize the CHW’ answers to questions about the COVID-19 impact on their work routine and on the provision of primary care by their FHS teams. Although the cities differed significantly in this aspect, all respondents believed their work routines were modified by the pandemic (n = 1,744, 90.27%), either strengthening (n = 731, 41.46%) or weakening (n = 783, 44.41%) the team spirit. Many reported reducing the frequency of home visits (n = 1,168, 60.55%), school health promotion activities (PSE) (n = 1,458, 75.66%), group-specific health promotion activities in the community (n = 1,788, 93.96%), and other on-site community services (n = 1,272, 66.01%) during the pandemic. The frequency of administrative activities remained unchanged for 896 CHW (46.94%). 

**Table 4:** Community health workers (CHW) perceived impact of COVID-19 on primary health care (PHC), related to the Family Health Strategy (FHS) work process, activities per city (N = 1,935).

	Fortaleza n (%)	João Pessoa n (%)	Recife n (%)	Teresina n (%)	Sobral n (%)	Juazeiro do Norte n (%)	Crato n (%)	Barbalha n (%)	p-value	Total n (%)
Impact of COVID on PHC										
Provision of primary care was affected [n = 1,818]										
No	14 (4.09)	19 (6.74)	18 (5.81)	30 (10.10)	15 (7.43)	12 (6.59)	11 (9.73)	5 (5.56)	0.122	124 (6.82)
Yes	328 (95.91)	263 (93.26)	292 (94.19)	267 (89.90)	187 (92.57)	170 (93.41)	102 (90.27)	85 (94.44)		1,694 (93.18)
Frequency of diabetes mellitus and arterial hypertension care [n = 1,934]										
No change	97 (26.65)	125 (41.39)	167 (52.02)	128 (41.42)	66 (32.51)	107 (49.77)	65 (51.18)	42 (45.16)	< 0.001	797 (41.21)
Lower	212 (58.24)	136 (45.03)	92 (28.66)	146 (47.25)	84 (41.38)	75 (34.88)	35 (27.56)	30 (32.26)		810 (41.88)
Higher	55 (15.11)	41 (13.58)	62 (19.31)	35 (11.33)	53 (26.11)	33 (15.35)	27 (21.26)	21 (22.58)		327 (16.91)
Frequency of antenatal care [n = 1,930]										
No change	255 (70.05)	175 (58.53)	229 (71.34)	136 (44.16)	154 (75.86)	119 (55.35)	93 (73.23)	66 (70.97)	< 0.001	1,227 (63.58)
Lower	55 (15.11)	81 (27.09)	45 (14.02)	137 (44.48)	20 (9.85)	57 (26.51)	9 (7.09)	8 (8.60)		412 (21.35)
Higher	54 (14.84)	43 (14.38)	47 (14.64)	35 (11.36)	29 (14.29)	39 (18.14)	25 (19.69)	19 (20.43)		291 (15.08)
Frequency of vaccination [n = 1,928]										
No change	231 (64.17)	164 (54.30)	221 (69.06)	138 (44.81)	114 (56.16)	76 (35.35)	70 (55.12)	41 (44.09)	< 0.001	1,055 (54.72)
Lower	48 (13.33)	64 (21.19)	47 (14.69)	121 (39.29	32 (15.76)	47 (21.86)	20 (15.75)	7 (7.53)		386 (20.02)
Higher	81 (22.50)	74 (24.50)	52 (16.25)	49 (15.91)	57 (28.08)	92 (42.79)	37 (29.13)	45 (48.39)		487 (25.26)
Frequency of tuberculosis care [n = 1,928]										
No change	246 (68.72)	221 (73.67)	250 (77.88)	155 (50.65)	159 (78.33)	134 (62.33)	94 (74.02)	73 (78.49)	< 0.001	1,332 (69.27)
Lower	96 (26.82)	67 (22.33)	41 (12.77)	143 (46.73)	34 (16.75)	70 (32.56)	25 (19.69)	17 (18.28)		493 (25.64)
Higher	16 (4.47)	12 (4.00)	30 (9.35)	8 (2.61)	10 (4.93)	11 (5.12)	8 (6.30)	3 (3.23)		98 (5.10)
Frequency of leprosy care [n = 1,922]									< 0.001	
No change	221 (62.08)	216 (72.24)	239 (74.45)	157 (50.97)	155 (76.35)	127 (59.07)	90 (70.87)	71 (76.34)		1,276 (66.39)
Lower	125 (35.11)	73 (24.41)	65 (20.25)	144 (46.75)	40 (19.70)	83 (38.60)	32 (25.20)	19 (20.43)		581 (30.23)
Higher	10 (2.81)	10 (3.34)	17 (5.30)	7 (2.27)	8 (3.94)	5 (2.33)	5 (3.94)	3 (3.23)		65 (3.38)
Frequency of infant care [n = 1,926]										
No change	76 (21.05)	75 (25.08)	135 (42.06)	71 (23.13)	16 (7.88)	49 (22.79)	42 (33.07)	25 (26.88)	< 0.001	489 (25.39)
Lower	271 (75.07)	203 (67.89)	169 (52.65)	216 (70.36)	182 (89.66)	159 (73.95)	74 (58.27)	60 (64.52)		1,334 (69.26)
Higher	14 (3.88)	21 (7.02)	17 (5.30)	20 (6.51)	5 (2.46)	7 (3.26)	11 (8.66)	8 (8.60)		103 (5.35)
Frequency of care for spontaneous demands [n = 1,930]									< 0.001	
No change	62 (17.08)	94 (31.33)	85 (26.48)	70 (22.73)	39 (19.21)	56 (26.05)	47 (37.01)	34 (36.56)		487 (25.23)
Lower	255 (70.25)	145 (48.33)	178 (55.45)	189 (61.36)	140 (68.97)	136 (63.26)	59 (46.46)	40 (43.01)		1,142 (59.17)
Higher	46 (12.67)	61 (20.33)	58 (18.07)	49 (15.91)	24 (11.82)	23 (10.70)	21 (16.54)	19 (20.43)		301 (15.60)
Frequency of family planning [n = 1,921]										
No change	49 (13.61)	107 (36.27)	114 (35.51)	72 (23.45)	81 (39.90)	70 (32.56)	51 (40.16)	45 (48.39)	< 0.001	589 (30.66)
Lower	308 (85.56)	183 (62.03)	200 (62.31)	224 (72.96)	112 (55.17)	138 (64.19)	72 (56.69)	43 (46.24)		1,280 (66.63)
Higher	3 (0.83)	5 (1.69)	7 (2.18)	11 (3.58)	10 (4.93)	7 (3.26)	4 (3.15)	5 (5.38)		52 (2.71)
Frequency of cervical cancer screening [n = 1,923]										
No change	31 (8.66)	75 (25.17)	41 (12.77)	74 (24.03)	26 (12.81)	40 (18.60)	46 (36.22)	25 (26.88)	< 0.001	358 (18.62)
Lower	325 (90.78)	216 (72.48)	275 (85.67)	227 (73.70)	174 (85.71)	174 (80.93)	79 (62.20)	66 (70.97)		1,536 (79.88)
Higher	2 (0.56)	7 (2.35)	5 (1.56)	7 (2.27)	3 (1.48)	1 (0.47)	2 (1.57)	2 (2.15)		29 (1.51)
Frequency of rapid testing [n = 1,922]										
No change	113 (31.39)	113 (38.18)	108 (33.64)	82 (26.71)	119 (58.62)	66 (30.70)	58 (45.67)	37 (39.78)	< 0.001	696 (36.21)
Lower	234 (65.00)	150 (50.68)	199 (61.99)	214 (69.71)	80 (39.41)	137 (63.72)	63 (49.61)	50 (53.76)		1,127 (58.64)
Higher	13 (3.61)	33 (11.15)	14 (4.36)	11 (3.58)	4 (1.97)	12 (5.58)	6 (4.72)	6 (6.45)		99 (5.15)
Frequency of pregnancy testing [n = 1,928]										
No change	195 (54.02)	139 (46.33)	160 (49.84)	100 (32.47)	143 (70.44)	74 (34.42)	71 (55.91)	53 (56.99)	< 0.001	935 (48.50)
Lower	144 (39.89)	142 (47.33)	142 (44.24)	184 (59.74)	45 (22.17)	127 (59.07)	41 (32.28)	34 (36.56)		859 (44.55)
Higher	22 (6.09)	19 (6.33)	19 (5.92)	24 (7.79)	15 (7.39)	14 (6.51)	15 (11.81)	6 (6.45)		134 (6.95)
Frequency of oral health appointments [n = 1,925]										
No change	35 (9.70)	73 (24.41)	44 (13.71)	38 (12.42)	28 (13.79)	50 (23.26)	32 (25.20)	16 (17.20)	< 0.001	316 (16.42)
Lower	318 (88.09)	213 (71.24)	267 (83.18)	252 (82.35)	168 (82.76)	152 (70.70)	88 (69.29)	68 (73.12)		1,526 (79.27)
Higher	8 (2.22)	13 (4.35)	10 (3.12)	16 (5.23)	7 (3.45)	13 (6.05)	7 (5.51)	9 (9.68)		83 (4.31)

Almost all respondents (n = 1,694, 93.18%) agree that the primary care services offered by the FHS team were negatively and significantly impacted during the pandemic, with considerable differences between cities. Cervical cancer screening (n = 1,536, 79.88%), oral health appointments (n = 1,526, 79.27%), infant care (n = 1,334, 69.26%), family planning (n = 1,280, 66.63%), spontaneous demands (n = 1,142, 59.17%) and rapid testing (n = 1,127, 58,64%) showed the greatest reduction in frequency. Table 4 shows more details for different FHS health care services per city. 

## Discussion

Our results reveal a substantial contribution of CHW, regulated as health professionals by *Law n. 14.536/2023* ^18^, in combating the COVID-19 pandemic as frontline workers within PHC. But we observed important differences between cities regarding training, the availability of PPE, the adoption of established biosafety procedures, and the perceived risk of workplace contamination. The participant CHW described a setting of physical vulnerability that led to feelings of fear, helplessness, and occupational insecurity. In all the cities, primary health care services were partially discontinued during the pandemic, drastically limiting population access to essential care. Understanding these aspects from the CHW’s perspective, who both act as a link between the community and the FHS teams and are particularly exposed to risks in the territory (they live and work in the same area), is crucial to evaluating the pandemic impact on PHC and to planning PHC activities, including preparedness for possible future pandemics.

The COVID-19 pandemic has impacted health systems worldwide, leading to interruptions in essential health programs and services, which persisted beyond the first year of the pandemic in approximately 90% of countries. These were sometimes associated with distrust from the community or fear of contamination at health care facilities, besides service-related issues such as insufficient personnel and resource allocation for complex emergencies, especially during the first wave ^19^.

In Brazil, first-wave response to COVID-19 focused on procuring ventilators, expanding the ICU capacity and hospital network for referral of severe patients, and adopting social distancing meausres ^20^ ^,^ ^21^. Despite the PHC ability to reduce transmission and identify and monitor cases within an organized care framework, the country lacked initial well-defined guidelines for primary health care professionals. The national guidelines ^9^ ^,^ ^15^ published represented an important step forward in this regard, but the federal government first concentrated its attention on hospital care rather than on PHC. Moreover, these publications made no mention of successful measures adopted in other countries, such as mass testing, quarantine, contact tracking, and lockdowns ^21^.

This may explain the heterogeneity between cities regarding PHC deployment for combating COVID-19. Apart the different population size, location, municipal government, socioeconomic profile, and infrastructure between the analyzed municipalities, other issues may have influenced the CHWs’ perception during the study period. This includes a lack of intergovernmental coordination associated with federal omissions regarding national management of the pandemic response ^22^, along with stances contrary to WHO recommendations based on contrastive scientific views and an emphasis on economic stability and fiscal austerity. In Brazil, state and municipal authorities undertook implementing pandemic control measures, horizontal cooperation strategies, and regional learning practices, reconfiguring inter-federative relations at the local and regional levels, considering the state’s competency in managing health policies as a means to find solutions to the challenges posed by the federal government ^22^. Knaul et al. ^23^ described this movement as “punt politics”, in reference to national federal leaders deferring or deflecting responsibility for health system decision-making to sub-national entities without evidence or coordination. This eased coordination issues, negatively impacting the health system resilience and COVID-19 in Brazil.

CHW and FHS work routines changed as facilities reduced individual and collective health promotion and disease prevention actions, and sometimes replaced them with administrative work. CHW actions and activities and the FHS teams were impacted differently depending on the city. School-based (PSE), community-based, and group-specific activities were more strongly affected than home visits and office work. PHC teams saw a drastic reduction in oral health services, cervical cancer screening, family planning, and infant care. Vaccination and antenatal care were the least affected activities during the pandemic. The discrepancies found between cities regarding these activities may result from the lack of clear federal guidelines concerning priorities. Different measures will be needed to recover “lost care” and services neglected in a range of scenarios. Strengthening the system to be more resilient is key to tackle present and future health threats.

Resilience building should be integrated with existing efforts to strengthen health systems ^24^. The pandemic has challenged local, national, regional and global capacities to prepare and respond, but the success of these strategies depends on how an existing health system is organized, governed and financed ^25^. During a crisis, resilient health systems are capable of effectively adapting in response to dynamic situations and reducing vulnerability across and beyond the system. CHW can play an important role as COVID-19 recovery agents for health systems and community resilience, as they navigate between vertical (state) and horizontal (community) forces, allowing them to interact with different people and affect (and be affected by) what happens in their community. This reinforces the interest in understanding, from the CHW perspective, the COVID-19 impact on PHC, and their ability to collaborate in the fight against COVID-19.

The COVID-19 pandemic also highlighted the need to reinforce health systems’ resilience to face future crises, which requires medium and long-term investments and transformations to ensure risk management capacity and the continuity of essential health services ^26^. In this perspective, the health system’s foundations, with a focus on PHC and on incorporating health security, must be strengthened.

Reports of deviations from the formal CHW job description over the past years increased during the pandemic with the added challenge of a lacking consensus between local and federal authorities regarding how to best combat the sanitary emergency in a context where denialist stances prevented these professionals from interacting with the population and carrying out their work, especially in low-income communities ^27^. Nevertheless, almost 80% of the respondents acted as frontline workers against the COVID-19 pandemic. It provided an opportunity to rethink and redirect their work under new forms of social behavior, including the adoption of professional instruments and strategies designed to meet the population’s health needs.

Changes to the work associated with protocol implementation and/or the re-dimensioning of team activities occurred, in most scenarios, without support from institutions of continuing education. This produced a mismatch between formal training and the actual tasks performed by CHW, as well as the expected impact from their work ^3^. Providing continuing health education in the pandemic context is very important for CHW, as is establishing a process of institutional support and supervision ^8^ ^,^ ^28^ ^,^ ^29^. Creating a forum of discussion on work organization would encourage health worker protagonism, strengthen the team spirit and enable co-management, favoring collective interests and the sharing of responsibility in public health care processes.

Combating the COVID-19 pandemic exposed CHW to the risk of infection and of transmitting the virus to coworkers and relatives. Inadequate working conditions, with insufficient PPE supply, lack of training in new protocols, and low levels of overall biosafety, may have contributed to the high number of participants who reported being infected or contaminating relatives and coworkers.

The picture of the CHW work our results paint helps to understand their occupational journey and the many challenges faced during the crisis, facilitating the identification of post-pandemic difficulties. The inter-city discrepancies observed reflect how different contexts were impacted by the pandemic and highlight the need for crisis preparedness in the form of multiple strategies, emphasizing the relevance of PHC for governance and the need for both inter-governmental coordination and locoregional sensitivity, strengthening the health system and its resilience. Adequate funding and political and human resources are fundamental to meet these needs, a challenge that has been discussed within the SUS since before the pandemic ^30^. PHC is one of the most cost-effective strategies for providing public health care, being less costly and technology-dependent than hospital-based strategies; to be fully functional and effective, however, it requires qualified professionals with occupational stability and adequate infrastructure. Investing in human resources is thus crucial, and implies implementing public policies for CHW’ continuing education and ensuring their working conditions (e.g., appropriate and sufficient PPE) so they can fulfill their tasks to improve health indicators in their territory. By strengthening PHC through health promotion, disease prevention, and surveillance, CHW have become an essential component of pandemic preparedness and protection. 

Despite the significant findings, the quantitative and cross-sectional sampling approach represented an important limitation. This method did not allow a longitudinal analysis of the PHC work reorganization associated with the pandemic and its impacts over time, nor an in-depth analysis of the CHW’ perception regarding the most relevant aspects influencing their work in the territory.

## Conclusions

Conducted with a sample of eight northeastern Brazilian cities of different sizes and profiles, this study revealed significant heterogeneity regarding the COVID-19 pandemic response measures, possibly associated with a lack of coordination by the Federal Government, as perceived by CHW. Regardless of the scenario, local health systems, workflows, services and PHC protocols employed by FHS teams had to be reorganized to meet the demand for safe care and service resilience. This requires dialogue, ample participation, and investments in continuing health education to ensure community access to quality services and proper guidance in case of sanitary emergencies. The importance of the SUS and its potential for reorganization during crisis must be acknowledged while preserving the headway made thus far.
